# Enhancement of Phenol Biodegradation by *Pseudochrobactrum* sp. through Ultraviolet-Induced Mutation

**DOI:** 10.3390/ijms16047320

**Published:** 2015-04-01

**Authors:** Zhen Mao, Chenyang Yu, Lingling Xin

**Affiliations:** School of Environment Science and Spatial Informatics, China University of Mining and Technology, Xuzhou 221008, China; E-Mails: bingyuer0120@163.com (C.Y.); xinlinglingcumt@163.com (L.X.)

**Keywords:** biodegradation, degradation kinetics, mutant, phenol, *Pseudochrobactrum* sp.

## Abstract

The phenol-degrading efficiency of *Pseudochrobactrum* sp. was enhanced by ultraviolet (UV) irradiation. First, a bacterial strain, *Pseudochrobactrum* sp. XF1, was isolated from the activated sludge in a coking plant. It was subjected to mutation by UV radiation for 120 s and a mutant strain with higher phenol-degrading efficiency, *Pseudochrobactrum* sp. XF1-UV, was selected. The mutant strain XF1-UV was capable of degrading 1800 mg/L phenol completely within 48 h and had higher tolerance to hydrogen ion concentration and temperature variation than the wild type. Haldane’s kinetic model was used to fit the exponential growth data and the following kinetic parameters were obtained: μ_max_ = 0.092 h^−1^, Ks = 22.517 mg/L, and Ki = 1126.725 mg/L for XF1, whereas μ_max_ = 0.110 h^−1^, Ks = 23.934 mg/L, and Ki = 1579.134 mg/L for XF1-UV. Both XF1 and XF1-UV degraded phenol through the ortho-pathway; but the phenol hydroxylase activity of XF1-UV1 was higher than that of XF1, therefore, the mutant strain biodegraded phenol faster. Taken together, our results suggest that *Pseudochrobactrum* sp. XF1-UV could be a promising candidate for bioremediation of phenol-containing wastewaters.

## 1. Introduction

Phenol, the simplest aromatic alcohol, is more acidic than alcohol, due to stabilization of the conjugate base through resonance in the aromatic ring. Since it was first extracted from coal tar in 1834, phenol has been widely used in the synthesis of organic chemicals, and has been continuously introduced into the aquatic environment through the effluent from coking plants, petroleum processing plants, chemical plants, pharmaceutical industries, *etc.* Because phenol may be harmful to aquatic ecosystems [1] and present a threat to humans through contamination of drinking water supplies [2], the removal of phenol has received considerable attention.

Phenol can be removed from effluents by physicochemical methods, such as ozonation [3], Fenton’s reagent [4], ultraviolet light (UV) [5], by the use of hydrogen peroxide [6], or by biological methods [7,8]. In general, biological treatment has advantages over physicochemical methods, as it requires fewer chemical agents and equipment, costs less, and results in less secondary pollution. Previous studies have investigated phenol biodegradation under both anoxic and aerobic conditions. Under aerobic conditions, phenol is initially oxidized by oxygenation into catechols, followed by the ortho- or meta-ring fission pathway [9,10]. The maximum tolerated concentration is 2400 mg/L [11]. Under anoxic conditions, phenol degradation is initiated via carboxylation followed by dehydroxylation and de-aromatization [12]. Because of a lack of suitable electron acceptors, it is difficult for microorganisms to degrade phenol under anaerobic conditions [13]. As the aerobic biodegradation of phenol has shorter lag phases and is more efficient than that under anaerobic conditions [14], the aerobic biodegradation of phenol should be studied more intensively. Most phenol-degrading microorganisms identified to date are members of the genus *Pseudomonas* [15,16], but other genera, such as *Bacillus* [9], *Acinetobacter* [17], *Sphingomonas* [18], *Paecilomyces* [19], *Trichosporum* [20], *Candida* [21], and *Penicillium* [22] have also been reported.

Mutations can be induced in genetic material by exposure to physical or chemical agents. UV irradiation is one of the physical strategies for inducing mutation by forming pyrimidine dimerization and cross-links in DNA [23]. In recent years, mutational approaches have been applied for the improvement of industrial strains, such as enhanced biodegradation of a sulfonated azo dye, green HE4B, in *Pseudomonas* sp. LBC1 [24], improved production of a heterologous amylase in the yeast *Saccharomyces cerevisiae* [25], and enhancement of polygalacturonase production by *Aspergillus* *sojae* [26]. These mutations resulted in changes in the production of microbial metabolites and enzymes, which can be applied in industry.

The present study was performed to isolate a bacterial strain that was able to efficiently degrade phenol and then to enhance its degradation ability by UV irradiation-mediated mutation. The growth and phenol degradation capacity and degrading enzymes of the isolated strain was examined and compared with the mutated strain. The overall purpose of this study was to demonstrate the effectiveness of and the pathway used for phenol degradation in the isolated strain and its mutant variant.

## 2. Results and Discussion

### 2.1. Isolation and Identification of the Strain

**Figure 1:**
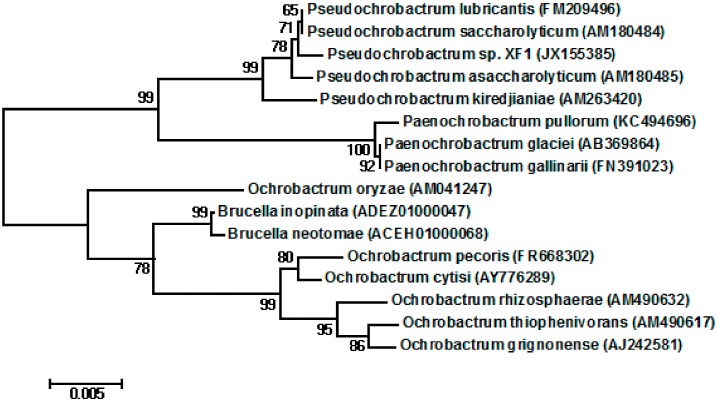
Phylogenetic tree showing relationships between *Pseudochrobactrum* sp. XF1 and related species. The tree was constructed from an alignment of the sequence of 16S rRNA using the neighbor-joining method. The number on the nodes represents the percentage of 1000 bootstrap replicates. The bar denotes the relative branch length. The numbers in brackets are GenBank accession numbers.

Bacterial strains were isolated from activated sludge from a local coking wastewater treatment plant. Only one strain survived and grew in the medium containing 1800 mg/L phenol (in our experiment); this strain, XF1, was selected for further studies. The physiological characteristics of *Pseudochrobactrum* sp. XF1 was compared with *Pseudochrobactrum lubricantis* KSS 7.8(T), which had the greatest sequence similarity to XF1 (Table 1). It was a rod-shaped, non-motile, gram-negative, catalase-positive bacterium capable of nitrate reduction. Glucose, mannose, fructose, *N*-acetyl-glucosamine, and galactose could be used as sole carbon sources. These characteristics of strain XF1 were similar to those of *Pseudochrobactrum* [27,28]. The partial 16S rRNA sequence of this bacterium, submitted to NCBI GenBank under accession No: JX155385, also highly resembled bacteria of the genus *Pseudochrobactrum*. Sequence similarities to the most closely related *Pseudochrobactrum* species were as follows: 99.8% similarity to the 16S rRNA sequence of *Pseudochrobactrum lubricantis* KSS 7.8(T), 99.7% to that of *Pseudochrobactrum saccharolyticum* CCUG 33852(T), 99.6% to that of *Pseudochrobactrum asaccharolyticum* CCUG 46016(T), and 99.0% to that of *Pseudochrobactrum kiredjianiae* CCUG 49584(T). We tentatively classified strain XF1 as a *Pseudochrobactrum* sp. The neighbor-joining tree is shown in Figure 1.

Existing data indicate that the species from the genus *Pseudochrobactrum* have potential application in Cr(VI) pollution bioremediation, such as *Pseudochrobactrum asaccharolyticum* LY6 [29] and *Pseudochrobactrum* sp. B5 [30]. *Pseudochrobactrum glaciale* was reported to be capable of producing ligninolytic enzymes [31]. To date, very few other industrially useful biological effects have been ascribed to this genus. In this experiment, we have shown that a species from the genus *Pseudochrobactrum* demonstrates phenol-degrading activities.

**Table 1:** Physiological characteristics of *Pseudochrobactrum* sp. XF1 and *Pseudochrobactrum lubricantis* KSS 7.8T.

Characteristics	Strain XF1	Strain KSS 7.8T
Morphology	rod-shaped	rod-shaped
Pigmentation	white	white
Motility	−	−
Gram reaction	−	−
Catalase	+	not detected
Nitrate reduction	+	+
Sugars assimilation:		
Glucose	+	+
Arabinose	−	−
Mannose	+	+
Fructose	+	+
*N*-Acetyl-glucosamine	(+)	+
Galactose	+	+

+, positive reaction; −, negative reaction; (+), weakly positive reaction.

### 2.2. Assay of UV Mutation

**Figure 2:**
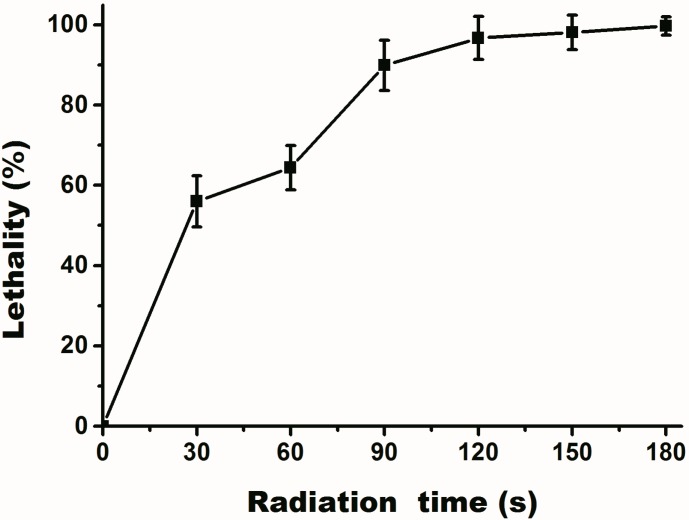
Death rate curve of *Pseudochrobactrum* sp. XF1 after different times of UV exposure. Error bars are the standard deviations.

UV was used for mutagenesis in this experiment. The results (Figure 2) demonstrate that *Pseudochrobactrum* sp. XF1 was sensitive to UV radiation. Lethality was increased with radiation time and reached 95% at 120 s, after which it stabilized. Thus, 120 s seemed to be a suitable radiation time for further studies. After screening for a strain that was able to degrade phenol more efficiently, we identified a strain that was used in further studies, and named it *Pseudochrobactrum* sp. XF1-UV. The phenol-degradation capability of *Pseudochrobactrum* sp. XF1-UV was stable for 20 continuous passages, demonstrating that the mutation was stably inherited.

### 2.3. Effect of pH and Temperature on Degradation of Phenol by Wild and Mutant Bacteria

**Figure 3:**
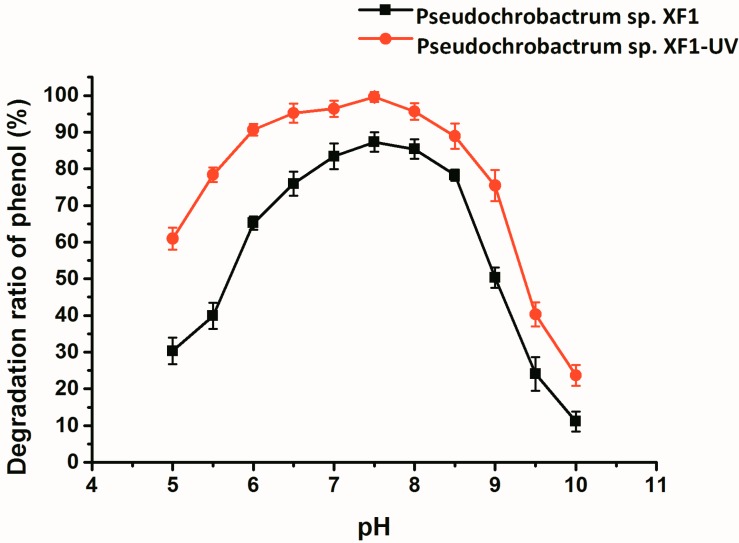
The effect of pH on phenol degradation by *Pseudochrobactrum* sp. XF1 and *Pseudochrobactrum* sp. XF1-UV. Error bars indicate standard deviations.

The degrading efficiency of wild and mutant bacteria at different pH values, varying from pH 5 to 10, is shown in Figure 3. The rate of phenol degradation was affected by the initial pH of the culture medium, with an initial phenol concentration of 1800 mg/L. The mutant strain demonstrated a higher phenol-degrading efficiency and wider optimum pH range than did the wild type. The highest phenol-degrading efficiency of both wild type and mutant cells occurred at pH 7.5. The phenol-degrading efficiency of the mutant was above 90% when the pH was in the range of 6.0–8.0. Compared with the mutant, the phenol-degrading efficiency of the wild type decreased significantly when the pH fell below 7.0 or rose above 8.5. These results indicated that the mutant strain has more tolerant over a wide range of pH values than the wild strain.

**Figure 4:**
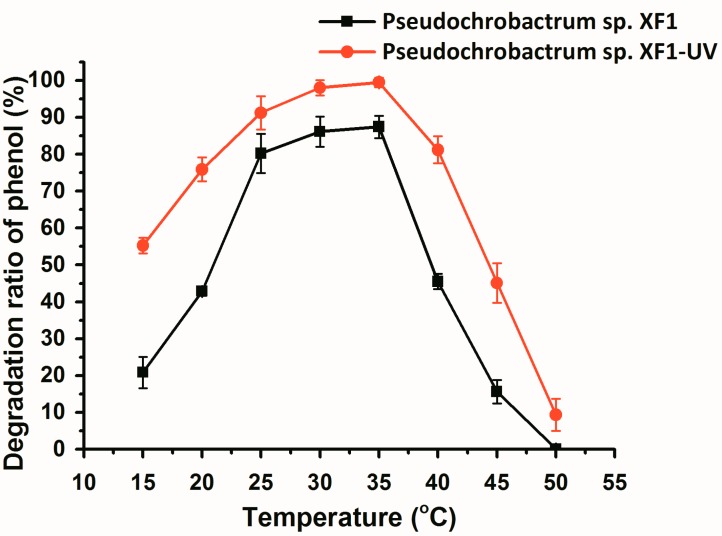
The effect of temperature on phenol degradation by *Pseudochrobactrum* sp. XF1 and *Pseudochrobactrum* sp. XF1-UV. Error bars indicate the standard deviations.

The effects of temperature on phenol-degrading efficiency were assessed at a temperature range of 15 to 45 °C, at pH 7.5, with a phenol concentration of 1800 mg/L (Figure 4). The phenol-degrading efficiency of both wild type and mutant cells did not change markedly when the temperature range was 25–35 °C. The phenol-degrading efficiencies of both wild type and mutant strains declined significantly at a temperature lower than 25 °C or higher than 40 °C. In particular, when the temperature was higher than 40 °C, the phenol-degrading efficiency of both strains declined sharply with the increase in temperature. When the temperature reached 50 °C, the wild type strain lost virtually all activity, and the mutant cells only retained weak activity. These data revealed that the ratio of phenol degradation was affected by temperature, particularly by high temperatures. Based on our findings, the mutant cells adapt more effectively to the changing environments (pH and temperature).

Many studies have isolated and characterized novel strains that are able to utilize phenol as the sole source of carbon and energy, at concentrations ranging from 100 to 2400 mg/L [20,21,32,33,34]. Only a few strains could completely degrade phenol within 48 h, with an initial concentration that exceeds 1800 mg/L [21]. Our data provide evidence that, after UV-induced mutation, the selected strain the phenol-degrading efficiency improved from 87.3% to 99.9%, with an initial concentration of 1800 mg/L phenol (Figure 4). Some papers reported that UV mutagenesis has a positive effect on increasing the biodegradation ability of strains [24,35]. Yet, another study showed that UV mutagenesis had no effect on degradation efficiency [36]. The enhanced phenol-degrading efficiency of mutant strains may result from the increase in the activity of the relevant enzymes; we therefore also studied the kinetics of phenol degradation.

### 2.4. Kinetics of Phenol Degradation

**Figure 5:**
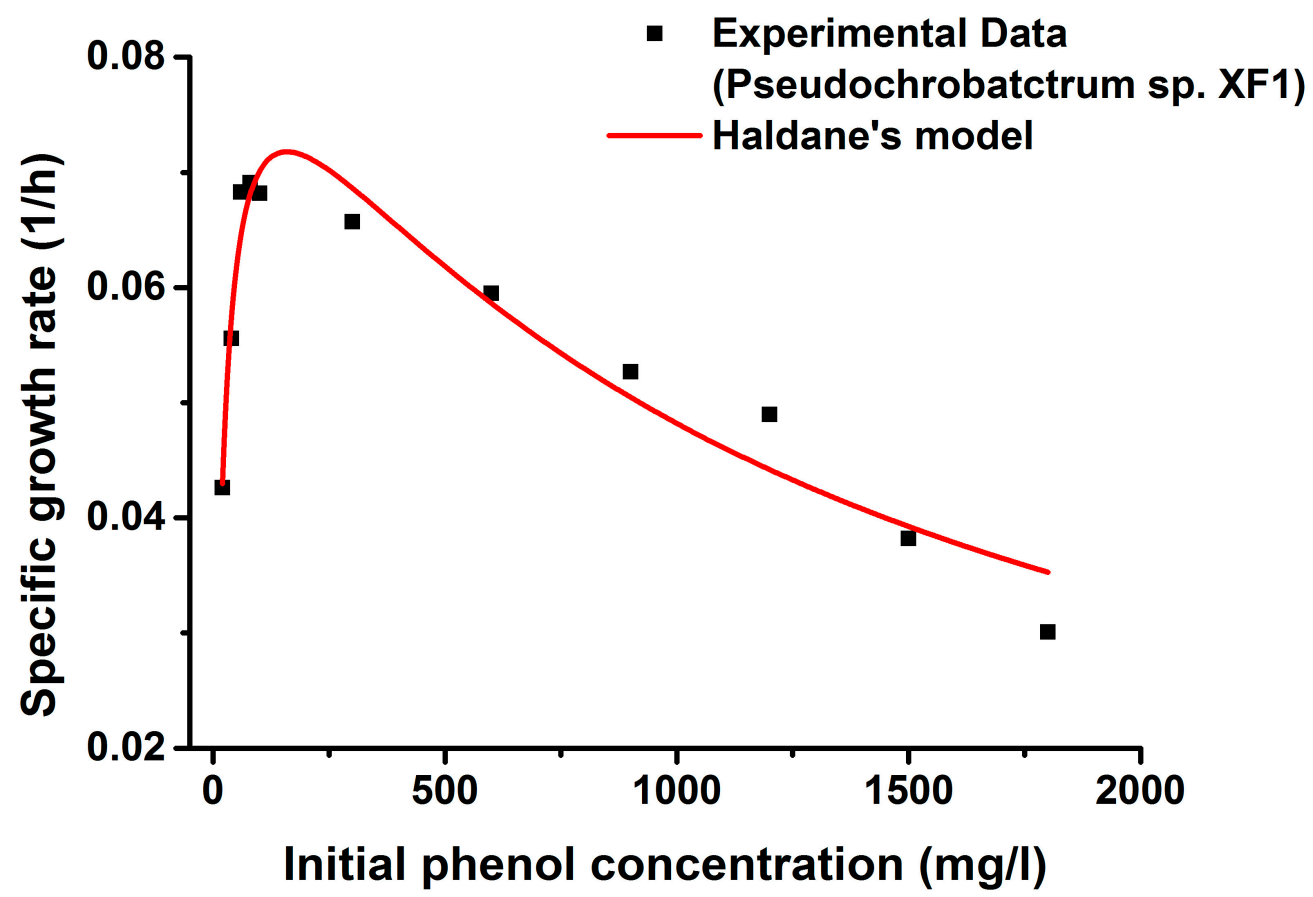
Experimental and predicted specific growth rates of *Pseudochrobactrum* sp. XF1 using Haldane’s model.

**Figure 6:**
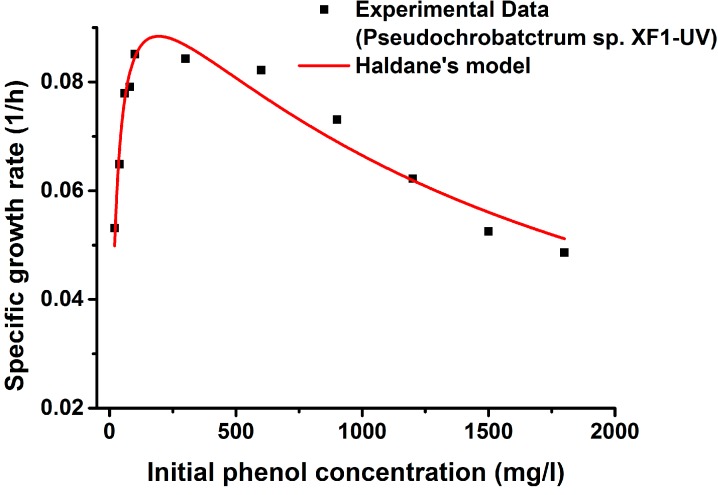
Experimental and predicted specific growth rates of *Pseudochrobactrum* sp. XF1-UV using Haldane’s model.

The specific growth rates of *Pseudochrobactrum* sp. XF1 and *Pseudochrobactrum* sp. XF1-UV with different initial phenol concentrations have been plotted in Figure 5 and Figure 6. The kinetic parameters (μmax, Ks, and Ki) were calibrated from experimental data in terms of Equation (1). As is shown in Figure 5 and Figure 6, the observed specific growth rate of both wild type and mutant strains increased initially with the increase in substrate concentration, up to a certain concentration for both bacterial strains, and subsequently tended to decrease with the increase in phenol concentration, owing to substrate inhibition. Kim *et al.* [37] showed that the decrease in the specific growth rate seemed to be strain-dependent and to be directly related to Haldane’s inhibition concentration (Ki). In our experiment, *Pseudochrobactrum* sp. XF1 showed a decrease at 80 mg/L, while *Pseudochrobactrum* sp. XF1-UV showed a decrease at 100 mg/L.

The experimental results fit Haldane’s kinetic model well. The estimated growth parameters of *Pseudochrobactrum* sp. XF1 were μ_max_ = 0.092 h^−1^, K_s_ = 22.517 mg/L, and K_i_ = 1126.725 mg/L (*R*^2^ = 0.938), whereas those of *Pseudochrobactrum* sp. XF1-UV were μ_max_ = 0.110 h^−1^, K_s_ = 23.934 mg/L, and K_i_ = 1579.134 mg/L (*R*^2^ = 0.939). Strain XF-1 and XF1-UV possessed significantly higher K_i_ values than most of the previously reported strains [9,16,19,32], even including *Rhizobium* sp. CCNWTB701 [38]. The higher K_i_ values of XF-1 and XF1-UV implied that the strains could tolerate higher substrate concentrations. The K_s_ value of XF1 was close to the value of XF1-UV, indicating that the inhibitory effects in XF1 and XF1-UV occurred at the same concentration. These results were consistent with the kinetic trends seen in the specific growth rate curves. In this study, strain XF1-UV present higher K_i_ and μ_max_ values than strain XF1, which suggested that XF1-UV has a higher tolerance and faster degradation ratio than XF1. Moreover, the tolerance characteristics of strains depended on their hereditary character [39]. In the present study, it could be due to the genetic modification in the activities of degradation enzymes by means of UV-induced mutation.

### 2.5. Activity of Enzymes in the Phenol Degradation Pathway

Under aerobic conditions, phenol was firstly monohydroxylated by phenol hydroxylase at the ortho position to the pre-existing hydroxyl group, and then this dihydroxylated intermediate was channeled into the ortho-cleavage pathway by catechol 1,2-dioxygenase (C12O), or into the meta-cleavage pathway by catechol 2,3-dioxygenase(C23O) [9,10]. Both types of pathways lead to intermediates of central metabolic routes, such as the tricarboxylic acid cycle. Strain improvement by mutation is a traditional method for isolating mutants producing enhanced enzyme levels [40]. In this study, the enzyme activities of phenol hydroxylase, C12O, and C23O were measured in both *Pseudochrobactrum* sp. XF1 and *Pseudochrobactrum* sp. XF1-UV (Table 2). Strains XF1 and XF1-UV demonstrated phenol hydroxylase and C12O activity, but C23O activity was not detectable in either strain. These results indicated that metabolism of phenol involves the ortho-pathway, which is consistent with the catabolic pathway in *Pseudomonas*, *Acinetobacter*, *Trichosporon*, and *Serratia* [41,42,43]. The phenol hydroxylase activity of strain XF1-UV was 2.5 times higher than that of strain XF1, while the C12O activity in strain XF1-UV was also a slightly higher than that of strain XF1. The results from the study by Zlatka *et al.* also showed higher phenol hydroxylase activities in the mutant than in the wild type strains [42]. Therefore, it seems that the genetic modifications by UV irradiation enhance the activities of the degrading enzymes, which can account for the higher efficiency of phenol degradation shown by strain XF1-UV.

**Table 2:** Enzymatic activities of phenol hydroxylase, catechol 1,2-dioxygenase, and catechol 2,3-dioxygenase from cell extracts of different isolates of the *Pseudochrobactrum* sp. XF1 and *Pseudochrobactrum* sp. XF1-UV strains.

Strain	Specific Activity (U·mg·Protein^−1^)		
	Phenol Hydroxylase	Catechol 1,2-Dioxygenase	Catechol 2,3-Dioxygenase
XF1	0.306 ± 0.009	0.324 ± 0.021	N.D.
XF1-UV	0.761 ± 0.053	0.392 ± 0.037	N.D.

All data shown were expressed as mean ± standard deviation (*n* = 3). N.D.: not detected.

## 3. Experimental Section

### 3.1. Chemicals and Culture Medium

The microorganism was grown in a basal mineral salt medium containing 0.5 g/L KH_2_PO_4_, 0.5 g/L K_2_HPO_4_, 0.2 g/L MgSO_4_·7H_2_O, 0.1 g/L CaCl_2_, 0.2 g/L NaNO_3_, 0.01 g/L MnSO_4_·H_2_O, and 1 g/L NH_4_Cl. This basal medium was supplemented with phenol, at different concentrations (100–1800 mg/L), as the sole carbon source. Mineral salts agar plates were prepared by the addition of 15 g/L agar to the liquid medium. The media were sterilized in an autoclave at 121 °C for 15 min. All chemicals were of analytical grade and were obtained from Sigma and Merck.

### 3.2. Selection and Isolation of the Phenol-Degrading Strains

Bacterial strains were isolated from activated sludge from a local coking wastewater treatment plant located in Xuzhou, Jiangsu Province, China. The activated sludge was serially diluted and plated onto mineral salts agar plates containing 100 mg/L phenol. Isolated colonies were re-streaked and the individual colonies were then transferred onto agar plates to assess their purity. Colonies were harvested from plates based on distinct colony morphology. Six colonies with distinct colony morphology were isolated from the media containing 300 mg/L phenol. These strains were further exposed to higher concentrations (up to 1800 mg/L) of phenol in liquid medium. Only one strain survived and grew in the medium containing 1800 mg/L phenol at 37 °C. This strain was named XF1 before its identification.

### 3.3. Identification of the Isolated Bacteria

The isolated strain was further characterized physiologically following standard procedures [44]. Morphology was characterized and motility was assayed using light microscopy (Olympus microscope CKX41, Tokyo, Japan). Then, genomic DNA was extracted from the isolated bacteria (XF1) according to protocols that had previously been described [45]. The extracted genomic DNA was used as a template for PCR. 16S rDNA was amplified by PCR using primers 357F (5'-CTC CTA CGG GAG GCA GCA G-3') and 1540R (5'-AGG AGG TGA TCC AGC CGC A-3'). The cycling profile was as follows: denaturation at 98 °C for 5 min; 35 cycles each consisting of 95 °C for 35 s, 55 °C for 35 s, 72 °C for 90 s, with a final elongation step at 72 °C for 8 min. PCR amplification is confirmed by agarose gel (1%) electrophoresis, followed by the purification step and sequenced by Sangon Biotech (Shanghai, China). The sequences obtained were compiled and compared with sequences in the GenBank database using BLAST. A maximum likelihood phylogenetic tree was generated with MEGA 5.1 using neighbor-joining methods with 1000 bootstrap trials.

### 3.4. Mutagenesis and Screening of Mutants

The isolated bacterial strain (XF1) was inoculated in a liquid medium at 30 °C for 24 h, after which the cells were inoculated in sterilized 0.2 M phosphate buffer (pH 7.0) at a density of 100 cells/mL. The cell suspension was irradiated with a UV lamp (254 nm, 30 W, at a distance of 40 cm) for 30, 60, 90, 120, 150, or 180 s, and then immediately sheltered from the light. The mutants were spread onto agar plates containing phenol (100 mg/mL). The plates were then incubated at 37 °C for 48 h, and were observed for colonies. All colonies were further exposed to higher concentrations (up to 1800 mg/L) of phenol in liquid medium. The mutant that was able to degrade phenol faster was named XF1-UV.

### 3.5. Biodegradation Experiments

The optimum temperature and pH for the degradation of phenol by strain XF1 and the mutant strain XF1-UV were determined. Each experiment was carried out in batch mode in 250-mL Erlenmeyer flasks containing 100 mL liquid media along with 1800 mg/L phenol and the cell suspension (OD: *ca.* 1.60). Effects of various pH values (5, 5.5, 6, 6.5, 7, 7.5, 8, 8.5, 9, 9.5, and 10) and temperatures (15, 20, 25, 30, 35, 40, 45, and 50 °C) were investigated after the cultures had been incubated for 48 h. All experiments were carried out in triplicate.

### 3.6. Phenol Degradation Kinetics

Experiments were performed at optimum environmental conditions using different initial phenol concentrations (20, 40, 60, 80, 100, 300, 600, 900, 1200, 1500, and 1800 mg/L), to investigate the effects of phenol concentration on cell growth [16]. Haldane’s kinetic model was selected to represent the degradation kinetics of phenol. The specific growth rate of strain XF1 or mutant strain XF1-UV, μ (h^−1^), was defined as:

(1) $$\text{μ}=\frac{{\text{μ}}_{\text{max}}\text{S}}{{\text{K}}_{\text{S}}+\text{S}+(\frac{{\text{S}}^{2}}{{\text{K}}_{\text{i}}})}$$

where μ is the specific growth rate (h^−1^), μ_max_, the maximum specific growth rate (h^−1^), S, the substrate concentration (mg/L), K_s_, the half-saturation constant of growth kinetics (mg/L), and K_i_ the inhibition constant (mg/L). The value of μ was determined at the exponential phase of the growth curve. From the linear plot of X *vs.* ln(Sx/S), after a short lag phase, the value of μ for an initial phenol concentration (S) is obtained, where X is the cell concentration in absorbance unit at an OD of 600 nm. From the values of μ *vs.* S, the values of μ_max_, K_s_, and K_i_ could be obtained using regression analysis in MATLAB 7.0. All the experiments were carried out under the optimum culture conditions for these strains.

### 3.7. Enzyme Assays

Cells were harvested in logarithmic phase from liquid medium and washed with 100 mM Na_2_HPO_4_–KH_2_PO_4_ buffer, pH 7.5, and ruptured by 30-s pulsed sonication (4 °C and six cycles). Cell debris was removed by centrifugation at 12,000× g at 4 °C for 40 min. The cleared supernatant was used for enzyme assays.

Phenol hydroxylase activity was measured spectrophotometrically by monitoring the decrease in absorbance at 340 nm [46]. C12O and C23O activities were determined spectrophotometrically by tracking the formation of cis,cis-muconic acid at 260 nm and of 2-hydroxymuconic semi-aldehyde at 375 nm, respectively [47,48]. Control reactions (without substrate or crude extract) were performed for each assay. One unit of activity was defined as the enzyme amount that was required to catalyze the formation of 1 μmol product per min at 25 °C. Specific activities of enzyme were expressed as units (U) per mg cell protein. The protein concentration was measured according to the Lowry method.

### 3.8. Analytical Methods

Cell and phenol concentration were analyzed as described by Banerjee and Ghoshal [9]. Cell density was monitored spectrophotometrically by measuring the optical density at 600 nm using a UV-spectrophotometer (Perkin Elmer, Norwalk, CT, USA). Aliquots (2 mL) of each sample was transferred to an Eppendorf tube and centrifuged at 12,000 rpm at 4 °C for 10 min. The pellet was discarded and the supernatant was collected for phenol quantification. The phenol concentration in the supernatant was measured using high-performance liquid chromatography (HPLC, Perkin Elmer, Norwalk, CT, USA). The supernatant was dissolved in distilled water to the desired concentration and then sterilized by filtration using 0.22-μm membrane filter, followed by HPLC analysis. HPLC was performed on a reverse phase C18 column (150 mm × 4.6 mm) with a methanol/water (60:40, *v*/*v*) mobile phase at a flow rate of 0.5 mL/min. Detection was performed with a UV detector (Perkin Elmer, Norwalk, CT, USA) at 270 nm. The concentration was measured using by comparing absorbance to a calibration curve.

## 4. Conclusions

In this study, a bacterial strain, XF1, which was capable of degrading phenol at a concentration of 1800 mg/L, was isolated from the activated sludge of a coking plant. The strain was identified as *Pseudochrobactrum* sp. based on 16S rDNA sequence alignment. Then, random UV-induced mutagenesis was used to improve its phenol-degrading efficiency. The mutant, XF1-UV, was capable of degrading phenol completely and had more tolerance to pH and temperature variation than the wild type strain. Furthermore, analysis of phenol-degradation kinetics also showed that XF1-UV has a higher tolerance and faster degradation ratio than XF1. Both the mutant and the wild type strain metabolized phenol through the ortho-pathway; however, the mutant was capable of producing higher levels of enzymes than the wild type strain. Therefore, our results demonstrate that *Pseudochrobactrum* sp. XF1-UV could be a promising candidate for biotreatment of phenol-containing wastewater or for bioremediation of phenol-contaminated sites. We are currently assessing the toxicity of samples after *Pseudochrobactrum* sp. XF1-UV treatment, to evaluate the potential of this organism for use in the bioremediation field.
